# Compositional and structural analysis of engineered stones and inorganic particles in silicotic nodules of exposed workers

**DOI:** 10.1186/s12989-021-00434-x

**Published:** 2021-11-22

**Authors:** Antonio León-Jiménez, José M. Mánuel, Marcial García-Rojo, Marina G. Pintado-Herrera, José Antonio López-López, Antonio Hidalgo-Molina, Rafael García, Pedro Muriel-Cueto, Nieves Maira-González, Daniel Del Castillo-Otero, Francisco M. Morales

**Affiliations:** 1 Pulmonology, Allergy and Thoracic Surgery Department, Puerta del Mar University Hospital, Cádiz, Spain; 2 Biomedical Research and Innovation Institute of Cádiz (INiBICA), Cádiz, Spain; 3 IMEYMAT: University Institute of Research in Electron Microscopy and Materials of the University of Cadiz, Puerto Real, Cádiz, Spain; 4 Department of Condensed Matter Physics, School of Sciences, University of Cádiz, Puerto Real, Cádiz, Spain; 5 Department of Anatomic Pathology, Puerta del Mar University Hospital, Cádiz, Spain; 6 INMAR: University Research Institute of Marine Research, University of Cádiz, Puerto Real, Cádiz, Spain; 7 Department of Physical Chemistry, CASEM, University of Cádiz, Puerto Real, Cádiz, Spain; 8 Department of Analytical Chemistry, CASEM, University of Cádiz, Puerto Real, Cádiz, Spain; 9 Department of Materials Science, Metallurgical Engineering and Inorganic Chemistry, School of Sciences, University of Cádiz, Puerto Real, Cádiz, Spain; 10 Department of Anatomic Pathology, Puerto Real University Hospital, Cádiz, Spain; 11 Department of Pulmonology, Puerto Real University Hospital, Cádiz, Spain

**Keywords:** Artificial stone, Engineered stone, Quartz agglomerate, Silicosis, Silica, Aluminum, Volatile organic compounds

## Abstract

**Background:**

Engineered stone silicosis is an emerging disease in many countries worldwide produced by the inhalation of respirable dust of engineered stone. This silicosis has a high incidence among young workers, with a short latency period and greater aggressiveness than silicosis caused by natural materials. Although the silica content is very high and this is the key factor, it has been postulated that other constituents in engineered stones can influence the aggressiveness of the disease. Different samples of engineered stone countertops (fabricated by workers during the years prior to their diagnoses), as well as seven lung samples from exposed patients, were analyzed by multiple techniques.

**Results:**

The different countertops were composed of SiO_2_ in percentages between 87.9 and 99.6%, with variable relationships of quartz and cristobalite depending on the sample. The most abundant metals were Al, Na, Fe, Ca and Ti. The most frequent volatile organic compounds were styrene, toluene and m-xylene, and among the polycyclic aromatic hydrocarbons, phenanthrene and naphthalene were detected in all samples. Patients were all males, between 26 and 46 years-old (average age: 36) at the moment of the diagnosis. They were exposed to the engineered stone an average time of 14 years. At diagnosis, only one patient had progressive massive fibrosis. After a follow-up period of 8 ± 3 years, four patients presented progressive massive fibrosis. Samples obtained from lung biopsies most frequently showed well or ill-defined nodules, composed of histiocytic cells and fibroblasts without central hyalinization. All tissue samples showed high proportion of Si and Al at the center of the nodules, becoming sparser at the periphery. Al to Si content ratios turned out to be higher than 1 in two of the studied cases. Correlation between Si and Al was very high (r = 0.93).

**Conclusion:**

Some of the volatile organic compounds, polycyclic aromatic hydrocarbons and metals detected in the studied countertop samples have been described as causative of lung inflammation and respiratory disease. Among inorganic constituents, aluminum has been a relevant component within the silicotic nodule, reaching atomic concentrations even higher than silicon in some cases. Such concentrations, both for silicon and aluminum showed a decreasing tendency from the center of the nodule towards its frontier.

## Introduction

Silicosis is an occupational respiratory disease produced by the inhalation of respirable crystalline silica; after disease onset, pulmonary fibrosis occurs, which can lead to respiratory failure and death [1]. It is a disease which incidence has been progressively reduced in recent years in countries with higher gross domestic product [2] due to the adoption of prevention measures and epidemiological surveillance, in addition to the displacement of mining activity to other countries with lower production costs. In the 1990s, the commercialization of a compound artificial material called quartz agglomerate, artificial stone or engineered stone (ES) began. This material is manufactured with finely crushed rock, with a silica content generally greater than 90%, together with pigments and polymeric resins [3] acting as binders of the inorganic portion. The design of this material and its attractive colors has made its use widespread in some countries as kitchen or bathroom countertops. Starting in 2009, cases of silicosis began to be described in workers who fabricate and install ES countertops, even producing two outbreaks focused in Israel [4] and Spain [5] and cases in countries such as Italy [6], the United States [7] and Australia [8], among others. In Australia, the significant number of cases detected has led to the consideration of ES related silicosis as an occupational epidemic [9]. One of the characteristics of ES silicosis is its short latency period and its aggressiveness [10], evolving to progressive massive fibrosis (PMF) in up to 37.7% of patients in a short period of time, even after having abandoned exposure to silica [11]. Several factors have been postulated as participants in the aggressiveness of this disease. One of them is the high silica content of which this material is composed, and another possibility is that the different components of ES, such transition metals or other elements, intensify the pulmonary fibrogenic response produced by silica [3, 6].

In 2009, an outbreak of ES silicosis began in our geographic area (the Bay of Cadiz, at the South of Spain), with a significant number of cases detected in subsequent years [5]. The objective of our study is to determine the different constituents that make up kitchen and bathroom countertops and to establish the localization of the deposit of metals and their atomic concentrations in lung tissue of ES workers diagnosed with silicosis. To address those goals, we have analyzed: (i) seven samples of those countertops with which patients more frequently worked during the years previous to their diagnosis; and (ii) lung biopsies samples, from the Anatomic Pathology files, of seven of those patients exposed to dusts of particles of these materials.

## Results

### ES samples

The first phase of the study consisted of analyzing the composition and structure of the composite materials with which the patients had worked during the years of exposure to ES. Samples were supplied by the Spanish National Association of Silicosis-Affected and -Sick People, having been selected among those most frequently utilized in the Bay of Cadiz region. The X-ray diffraction (XRD), X-ray fluorescence (XRF) and inductively coupled plasma mass spectrometry (ICP-MS) results for samples M1-M7 are included in Table 1.

**Table 1:** Percentages of crystalline phases measured by XRD, atomic percentage of more often elements measured by XRF, and trace elemental analyses of specific metal species by ICP-MS

Sample/method	M1	M2	M3	M4	M5	M6	M7
**XRD** (cryst. %)	2% Qu 98% Cri	25% Qu 45% Cri 30% Alb	100% Cri	53% Qu 47% Cri	56% Qu 44% Cri	100% Qu	100% Qu
**XRF** at. %*	66.6 O 32.7 Si 0.5 Ti	64.5 O 29.3 Si 3.3 Na 2.5 Al 0.2 Ti 0.1 Ca	66.7 O 32.7 Si 0.6 Ti	66.6 O 33.2 Si 0.1 Ti	66.7 O 33.2 Si	66.6 O 32.9 Si 0.3 Ti	64.4 O 30.4 Si 3.3 Na 1.4 Ca 0.1 Mg
ICP (mg/kg)**							
As	< 0.2	< 0.2	< 0.2	< 0.2	< 0.2	< 0.2	< 0.2
Cd	0.014	0.007	0.006	< 0.003	< 0.003	0.006	0.015
Co	42.2	62.4	29	127	102	75	71.3
Cu	4.93	13	5.30	98.1	50.2	58	96.0
Ni	5.21	9.1	3.21	21.6	16.4	12	12.4
Pb	1.1	1.95	1.2	0.53	0.66	2.21	409
Zn	3	20.5	6.56	256	59.5	228	312
Ba	24	16	25	11	10.3	13.4	262
Mo	0.222	0.251	0.204	0.385	7.44	0.280	0.413
Sb	0.151	0.150	0.154	0.150	0.170	0.154	0.413
V	0.971	1.72	0.870	0.592	208	0.830	2.20
Ti	8992	2861	9527	1067	691	4228	614
Al	1730	43,000	1622	1439	1900	1755	536
Ca	124	3188	115	152	169	143	45,546
Na	1913	30,250	2054	1216	1437	351	38,281
Fe	250	334	235	506	400	523	670
W	281	564	156	1221	1015	695	705

^*^Measurements < 0.1% have been discarded

^**^Limits of detection (mg/kg): As = 0.2, Cd = 0.003, Co = 0.230 Cu = 0.120, Ni = 0.084, Pb = 0.240, Zn = 0.580, Ba = 0.360, Mo = 0.130, Sb = 0.060, V = 0.130, Al = 100, Fe = 10, Ca = 20, Na = 110, Ti = 4, W = 30

*Qu* quartz, *Cri* cristobalite, *Alb* albite, *XRD* X-ray diffraction, *XRF* X-ray fluorescence, *ICP* Inductively coupled plasma

Regarding the different polymorphic phases of crystalline silica (SiO_2_) detected by XRD, samples M6 and M7 are formed exclusively of quartz, and samples M1 and M3 are almost fully, or completely, based on cristobalite, respectively. The rest of samples showed variable mixtures of cristobalite and quartz, with a remarkable portion assignable to albite (NaAlSi_3_O_8_) in one of the samples (M2). Considering the mostly present elements in the XRF studies (only those over 0.1 at.% are considered), O and Si were measured in percentages that agree well with the 2:1 ratio corresponding to SiO_2_. The XRD observed presence of albite in M2 is also in agreement with the XRF results (for a slightly Ca-doped albite). Since XR fluorescence (microanalysis) focus in a much smaller region of the sample than XR diffraction (more macroscopic analysis), probably a similar tectosilicate of the feldspar mineral group consisting in a solid solution of albite and anorthite endmembers (NaAlSi_3_O_8_–CaAl_2_Si_2_O) was probed in the portion of the sample M7 tested by XRF, considering the elemental quantifications.

Note that additional metallic elements were detected in smaller concentrations by XRF for which their quantifications would not be trustable, so focused analyses to these trace species were performed by ICP-MS (As, Cd, Co, Cu, Ni, Pb, Zn, Ba, Mo, Sb, V) or ICP-AES (Al, Fe, Ca, Na, Ti, W), as well as for other metals that are known to be harmful. The concentrations of these metals were variable between the different types of ES samples. The most abundant elements were Al, Na, Fe, Ca, Ti and W, some of which showed concentrations above 1 g/kg in some of the samples. Elements such as Co, Cu, Ni, Zn and Ba were present in variable concentrations between 3 and 312 mg/kg. The metals considered more toxic, such as As, Cd, or Pb, were detected at concentrations below 0.2 mg/kg, except Pb, that ranged from 0.53 to 409 mg/kg. Other elements, such as Mo, Sb and V, were also present but in lower concentrations.

Based on the gas chromatography coupled to-mass spectrometry (GC–MS) results (Table 2), the M5 sample had the highest concentration of volatile and semivolatile organic compounds (VOCs), i.e., 3.21 mg/kg for the volatiles considered. The common compound in all samples was styrene, while m-xylene and toluene were found in 4 of the samples. Notably, organic compounds not included in the analysis (non-target, Nt) were identified in all samples, with sample M5 showing the highest number (5 Nt in total). With respect to polychlorinated biphenyls (PCBs) and polycyclic aromatic hydrocarbons (PAHs), PCBs were not detected in any of the samples analyzed, and among the PAHs, phenanthrene, naphthalene, fluorene and fluoranthene stood out in the different samples. The sample that showed the highest concentration of PAHs was M7, with a value of 95.8 ng/g, and M2 showed the greatest number of different compounds (9 in total), some of which were below the limit of quantification or at the limit of detection.

**Table 2:** Concentrations of organic compounds (analyzed by GC–MS*)

Sample	M1	M2	M3	M4	M5	M6	M7
VOCs^†^ (mg/kg)	STY: 0.81 BAL: Nt PA: Nt BA: Nt	STY: 0.79 BAL: Nt PA: Nt TCS: Nt	STY: 0.76 2EH: 0.87 PA2DM: Nt BAL: Nt PA: Nt TCS: Nt	STY: 0.83 mX: 0.71 TOL: 0.75 2EH: 0.9 TMA: Nt BAL: Nt PA: Nt TCS: Nt	STY: 0.81 mX: 0.73 TOL: 0.79 2EH: 0.88 BAL: Nt PA: Nt PA2DM: Nt DPG: Nt TCS: Nt	STY: 0.74 mX: 0.85 TOL: 0.80 BAL: Nt PA: Nt	STY: 0.88 mX: 0.71 TOL: 0.71 BAL: Nt PA: Nt BA: Nt
PAHs^‡^ (ng/g)	ACY: LOD ANT: ND BaA: ND CH: LOQ FLA: 9.2 FLU: 18.0 NAP: 10.8 PHE: 17.7 PYR: ND	ACY: LOD ANT: 18.5 BaA: LOQ CHR: LOQ FLA: 9.4 FLU: 18.8 NAP: 22.9 PHE: 20.8 PYR: LOQ	ACY: LOD ANT: LOD BaA: ND CHR: LOD FLA: ND FLU: 17.0 NAP: 14.4 PHE: 17.8 PYR: LOQ	ACY: LOD ANT: LOD BaA: ND CHR: LOQ FLA: ND FLU: 17.1 NAP: 15.3 PHE: 18.2 PYR: ND	ACY: LOD ANT: ND BaA: ND CHR: LOD FLA: ND FLU: ND NAP: 14.7 PHE: 19.1 PYR: LOQ	ACY: LOQ ANT: ND BaA: ND CHR: LOQ FLA: ND FLU: 16.5 NAP: 9.3 PHE: 21.6 PYR: ND	ACY: LOQ ANT: ND BaA: ND CHR: LOQ FLA: 9.5 FLU: 18.3 NAP: 39.3 PHE: 28.8 PYR: LOQ

^*^ GC–MS: Gas chromatography-mass spectrometry

^†^ VOCs: Volatile organic compounds

^‡^ PAHs: Polycyclic aromatic hydrocarbons

*2EH* 2-ethyl-1-hexanol, *BAL* benzaldehyde, *BA* benzyl alcohol, *PA2DM* propanoic acid2,2 dimethyl, *mX* m-xylene, *PA* phthalic acid, *TMA* trimethylacetic acid, *STY* styrene, *TOL* toluene, *DPG* dipropylene glycol, *TCS* triclosan, *ACY* acenaphthylene, *ANT* anthracene, *BaA* benzanthracene, *CHR* chrysene, *FLA* fluoranthene, *FLU* fluorine, *NAP* naphthalene, *PHE* phenanthrene, *PYR* pyrene, *Nt* (nontarget) compounds detected (NIST Database) but not quantified, *ND* not detected, *LOD* limit of detection, *LOQ* limit of quantification

### Patients and biopsy samples

Patients were all male and they had been working cutting and polishing slabs of ES in small factories and in-home installation of ES countertops. Their ages ranged from 26 to 46 years (mean ± SD: 35.8 ± 6.5) and the exposure took place during periods of time from 10 to 23 years (mean ± SD: 13.7 ± 4.6), as presented in Table 3.

**Table 3:** Sociodemographic and epidemiological data

Patients	Age*	Year*	Duration of exposure (years)	Latency^†^	Smoking status^*, ‡^	pack-years*	Type of lung biopsy^ξ^
S1	26	2010	10	10	S	10	TBB
S2	31	2010	15	16	NS	-	TBB
S3	33	2010	10	11	S	1	TBB
S4	37	2010	10	10	NS	-	TBB
S5	37	2010	13	13	S	3	TBB
S6	41	2018	15	18	NS	-	VATS
S7	46	2016	23	23	Ex	25	VATS

^*^ At the time of lung biopsy

^**†**^Latency period (years) from onset of exposure to biopsy

^**‡**^S: Smoker. NS: Non-Smoker. Ex: Ex-smoker

^ξ^TBB: Transbronchial biopsy. VATS: Video-assisted thoracoscopic surgery

Samples of the lung were obtained using transbronchial biopsy (BTB) for five of the patients, and via video-assisted thoracoscopic surgery (VATS), in the case of the other two patients. Afterwards, the so-obtained biopsies were conserved in paraffin blocks, that allowed further processing.

Through tomographic cuts, histological samples (identified with the same label of the corresponding patient, as indicated in Table 3) were achieved, revealing the following patterns via light microscopy: well-defined silicotic nodule with a paucicellular lamellar fibrohyaline center and surrounded by a macrophagic population (S7), well-defined densely collagenized nodules alternating with other nodules consisting of fibroblasts and histiocytes without hyalinization (S6), well or ill-defined nodules composed of histiocytic cells and fibroblasts with some infiltration of the pulmonary interstitium by histiocytes and fibroblasts (S2–S5) or patchy interstitial infiltration consisting of fibroblasts and histiocytes reaching the alveolar septa and thickening them (S1). No one of these nodules (S1–S5) showed hyalinization.

**Fig. 1:**
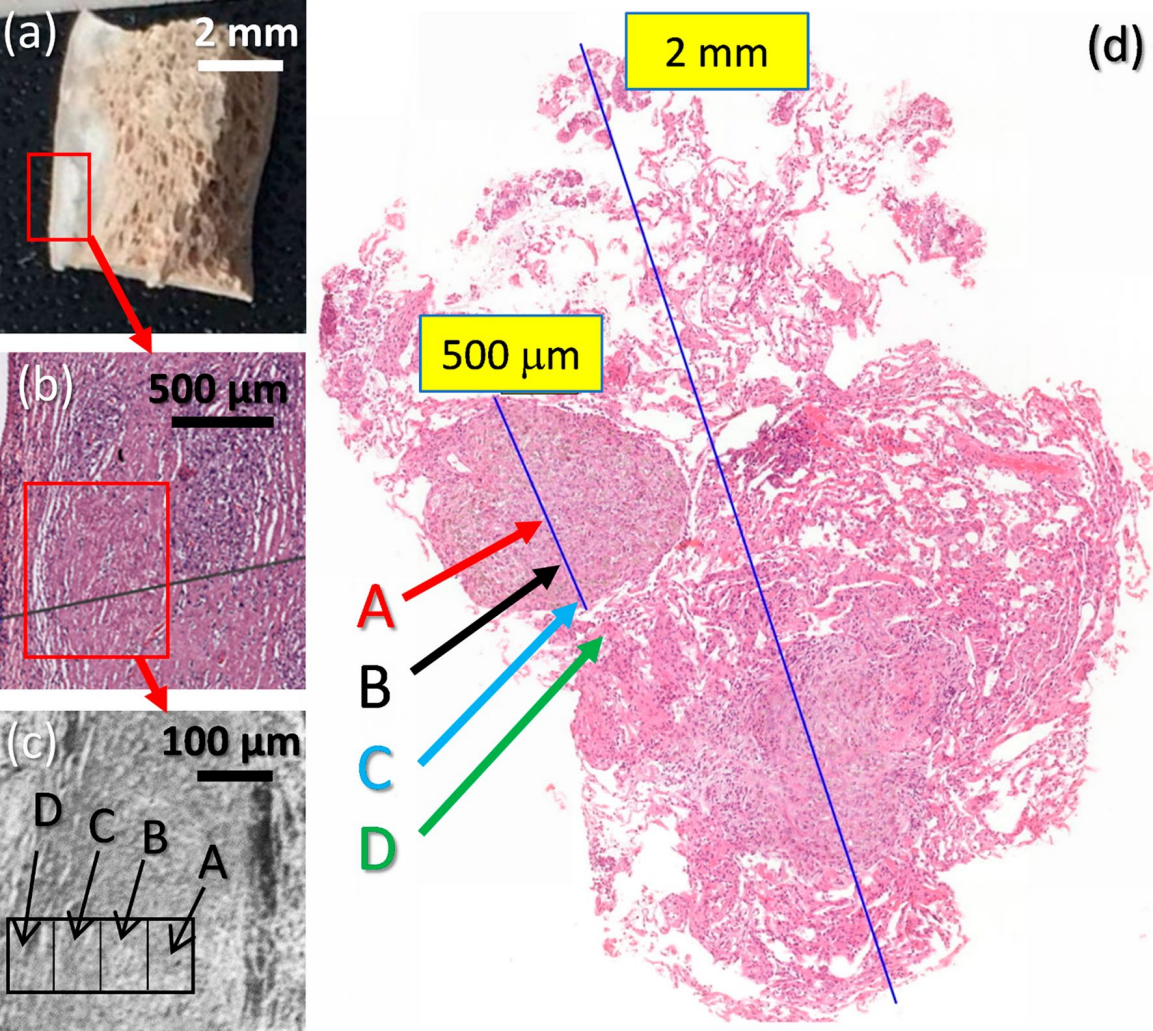
Photographic image (**a**) and optical (**b**) and SEM (**c**) micrographs of sample S7, indicating an area surrounding a silicotic nodule close to the visceral pleura. In the SEM image, regions A to D are indicated. Histological overview of sample S3 showing the optical micrograph of a biopsy containing a well-defined nodule composed of histiocytic cells and fibroblasts, with indication of positions defined from A to D in the present study (**d**)

Samples were then prepared for a nanoscale examination via scanning electron microscopy (SEM) related techniques. To this end, the biopsy samples were first prepared so the biological tissue could be studied under the electron beam. In this work, four main areas (regarding the silicotic nodules) for each biopsy samples were analyzed using SEM: one region in the center of the nodule, an area separated of such center, a third region close to the border of the nodule, and a fourth region practically outside of the nodule. These regions of study are referred with the labels “A”, “B”, “C” and “D”, respectively, throughout this work. Figure 1 shows a photograph (Fig. 1a) of one of the samples, S7, indicating the place closer to the optical micrograph (Fig. 1b) obtained for the studied nodule. Over this image, a red square indicates the area around the nodule from which a low-magnification SEM image is recorded (Fig. 1c), indicating in such image those four areas (A to D) that have been detailed studied. It is worth indicating that, while samples S6 and S7 sizes were in the range of 0.5 to 1 cm (as can be observed in Fig. 1a), samples S1 to S5 sizes were not larger than 2 mm, which made the micromanipulation of these samples a difficult process.

**Fig. 2:**
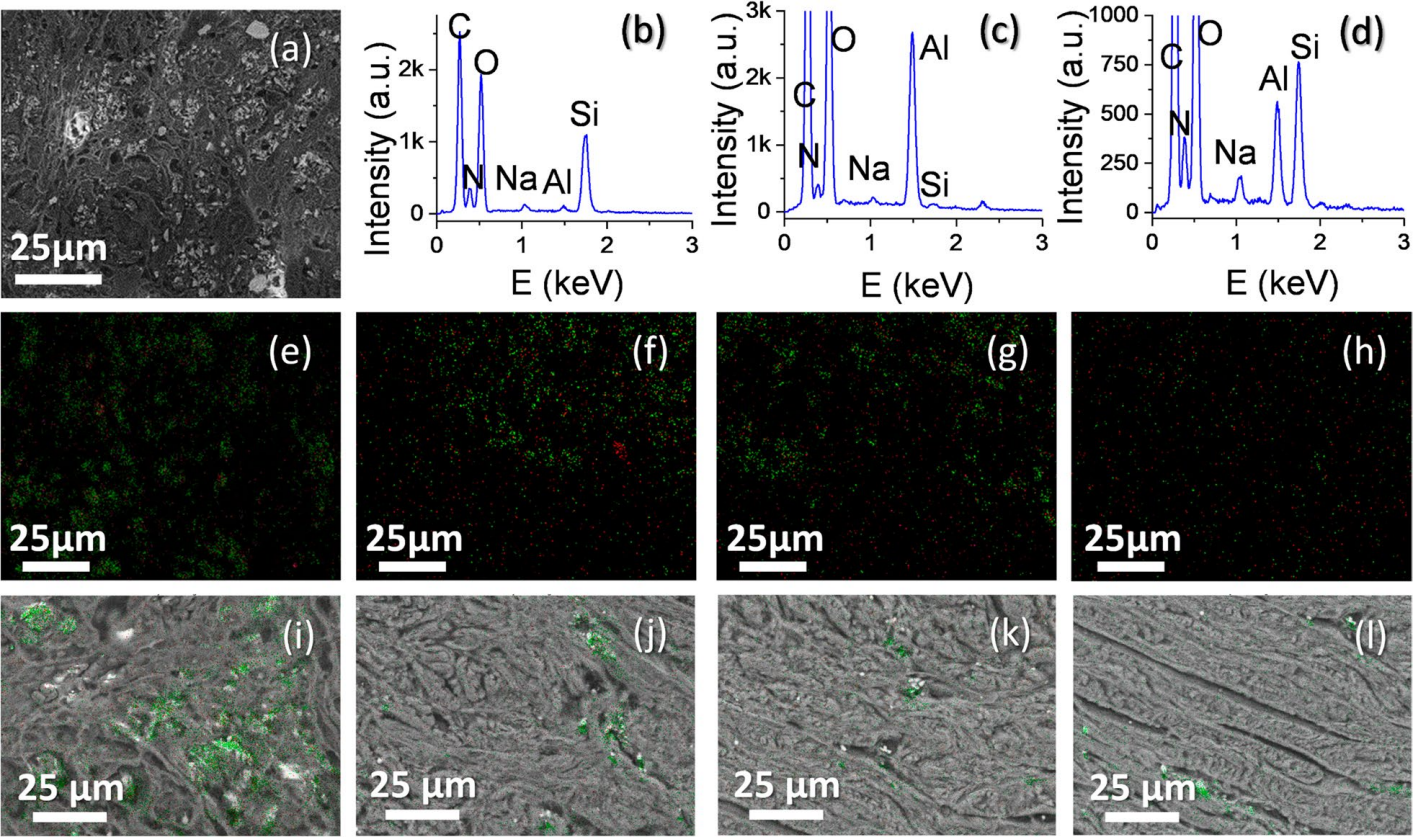
BSE-SEM micrograph (× 1000 magnification) from the center of a nodule in sample S3 (**a**), together with representative EDX map from regions A (**e**), B (**f**), C (**g**) and D (**h**) for Si (green) and Al (red) signals. Overlay of BSE-SEM images and EDX maps for Si (green) in sample S7 for regions A (**i**), B (**j**), C (**k**) and D (**l**)

Such regions A to D were systematically studied for each sample, using SEM images of the same area (8.65 μm^2^, that is, a × 1000 magnification image). Figure 2 presents representative results on this SEM study, which led to the data collected in Table 4.

**Table 4:** EDX measured for atomic contents of Si, Al and others (C, O, N, Na, P, S) for regions A to D from all biopsy samples

Sample	Region	Si (%)	Al (%)	Others (%)
**S1**	A	0.11	0.14	99.75
	B	0.08	0.03	99.91
	C	0.06	0.03	99.89
	D	0.03	0.01	99.96
**S2**	A	1.81	0.44	97.75
	B	0.44	0.14	9.42
	C	0.11	0.04	99.85
	D	0.02	0.04	99.94
**S3**	A	2.09	0.68	97.23
	B	1.32	0.24	98.44
	C	1.04	0.24	98.72
	D	0.08	0.07	99.85
**S4**	A	2.91	0.79	96.3
	B	0.83	0.18	98.99
	C	0.55	0.16	99.29
	D	0.12	0.10	99.78
**S5**	A	0.08	0.12	99.80
	B	0.05	0.11	99.84
	C	0.05	0.10	99.85
	D	0.02	0.02	99.96
**S6**	A	0.18	0.14	99.68
	B	0.08	0.43	99.49
	C	0.08	0.12	99.80
	D	0.00	0.00	100.00
**S7**	A	0.96	0.10	98.94
	B	0.08	0.09	99.83
	C	0.03	0.07	99.90
	D	0.04	0.01	99.95

Since SEM secondary electron images offer no compositional contrast, it may be difficult to even distinguish a silicotic nodule from healthy tissue using this type of signal. Therefore, as a first step, optical micrographs were utilized as guidance, in order to realize the approximate region where these nodules were. Next, at a higher magnification, SEM images formed by backscattered electrons (BSE) were utilized to locate the center of the nodules. This is an effective methodology since BSE images are composition sensitive, and thus, can be used as a way to reveal metal-containing particles (such as the ones consisting in different minerals formed by silicon or aluminum). Figure 2a shows a BSE image of the center of a nodule in sample S3, where the image contrast reveals a large number of micrometric particles of different composition to the organic background. These types of images show a high density of exogenous particles, presumably consisting in different minerals from the inhaled dust. Nevertheless, these images do not give more precise information on the composition of those particles, and, even, the bright regions in such images could be due to electrical charge accumulation at non-conducting areas. Thus, a spectroscopic technique had to be applied. In this sense, the energy dispersive X-ray spectroscopy (EDX) detector of the SEM was utilized in order to obtain point spectra from different particles (such as the ones presented in Fig. 2b to d), as well as map spectra from whole areas (Figs. 2e to l). When point spectra from individual particles are recorded, most particles in nodules revealed a signal similar to the one presented in Fig. 2b, where a clear Si-peak indicates (most probably) that the particle is formed by silica. In few cases, corresponding mostly to the central areas of nodules, spectra like the ones in Fig. 2c were detected. These spectra, with an Al-peak not accompanied by a Si-peak, might be associated to alumina particles, most probably. Figure 2d, on the other hand, reveals a more frequent spectrum than the ones presented in Fig. 2c, but not so frequent than the spectrum in Fig. 2b. This case presents both Si and Al peaks, and thus can be associated to different minerals containing both elements. It is worth mentioning that, although particle spectra such as the ones in Fig. 2b are detected in all regions around the nodule (regions A to D), signals of Al (Fig. 2c, d) are only detected on regions closer to the center of the nodule (regions A and B).

Beside the study of particle distribution and composition, the composition of whole regions A to D for each sample were obtained, using EDX maps of all areas. Figure 2e–h presents a representative result of EDX maps for Si (green) and Al (red) signals for regions A, B, C and D, respectively, in sample S3. It can be observed that the center of the nodule is highly populated by Si-containing particles, and such particle density decreases for regions further to this center. Particles with Al content (with or without Si) were mostly found at regions A and B. We found out that all the samples (S1 to S7) showed a similar trend (decreasing Si from regions A to D; Al mostly presented in regions A and B, only differing in the density of Si or Al containing particles, as will be more clearly seen from data in Table 4.

These EDX maps can be overlaid to BSE images, in such a way that it is possible to distinguish which of the bright regions in the BSE images correspond to Si(Al)-containing particles from those bright regions produced by charge effects in the SEM image. An example of this is presented in Fig. 2i to h, which correspond to areas A to D, respectively, for sample S7.

**Fig. 3:**
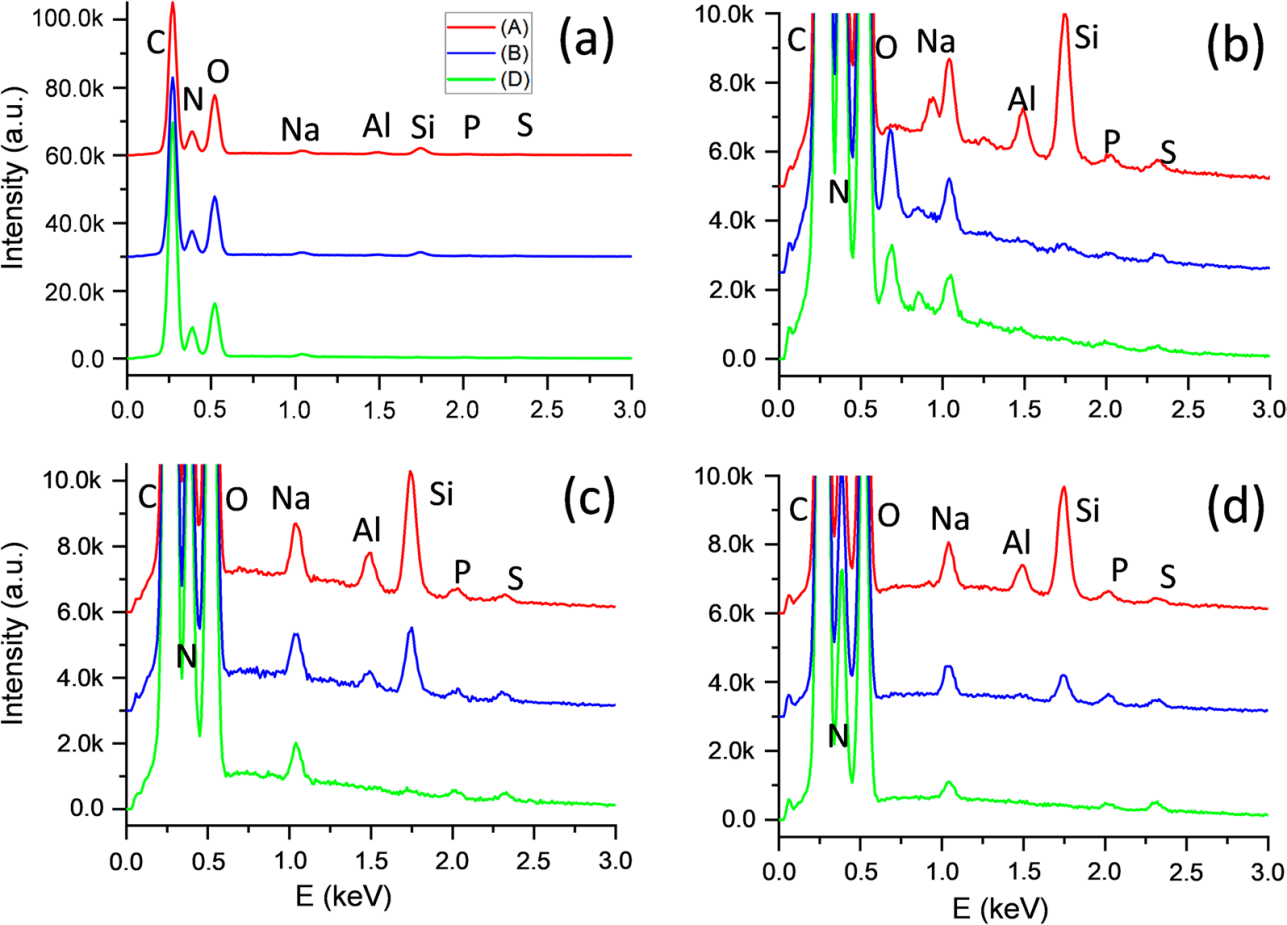
EDX spectra normalized to the N peak for samples S2, S3 and S4, obtained from regions A (red), B (blue) and D (green) of a nodule for samples S3 (images (**a**) and (**c**)), S2 (**b**) and S4 (**d**)

In order to quantify the content of Si and Al along the nodules, for every sample, EDX spectra were taken from each region (A to D). These integrated spectra (of which examples are presented in Fig. 3, for 3 samples and regions A, B and D, having normalized the spectra to the N-peak consisted, in each case, in collecting the X ray signal coming out of each the volumes formed by the 8.65 μm^2^ area and the length the electron beam extracted the X ray signal (about 1 µm, although the exact depth depends also on the material interacting with the electron beam). Thus, most of the amount of elements detected in these integrated spectra consisted in C, O and N, as main constituents of organic materials. Other elements found in the spectra (Na, P and S) were also components of the lung tissue. This fact lowers the percentage of Si and Al detected at each one of the regions. In this sense, Fig. 3a shows that when all peaks are fully visible, it is difficult to distinguish Al and Si peaks. Therefore, Fig. 3b–d presents a zoomed vertical ranged of these spectra, allowing a clear visualization of the already commented trend for Al and Si: a higher content at center of the nodule, and lower by the border. Note that two unidentified peaks appear for regions B and D in Fig. 3b, at about 0.69 keV. These artefacts, called “sum peaks”, are produced when C and N characteristic X-rays reach the spectrometer spaced by times that are shorter than the detector dead time, and thus, the signals are identified as corresponding to an energy which is sum of those of the C and the N. Therefore, those peaks, which are not associated to other elements, are to be disregarded.

The quantification of the elements in each sample and region was obtained from those spectra, and is collected in Table 4. In this table, the commented trend is stablished: The Si percentage was higher at the central zone of each nodule and lower towards the frontier of the nodule, while the same occurred with aluminum, which was found mainly around the central region, becoming sparser in the periphery. In all samples but one (S6), Si and Al were detected outside the nodule, in region D, although in minor atomic concentrations.

There are statistically significant differences in the distribution of Al and Si atomic contents among all the areas of the nodules (A to D, *p* < 0.05). All post-hoc comparisons in these cases are significant, so these differences were present when comparing one location with any other. It is noteworthy the difference in A compared to the rest of the areas, where the atomic contents of Si and Al are much higher.

In a parallel preparation (not shown here) of a lung sample from a control patient, who was not professionally exposed to silica, no silicon or aluminum was detected, and only the most abundant components of the human body were found (sodium, phosphorus, sulfur, oxygen, nitrogen and carbon).

Radiological classification and progression are shown in Table 5. Five patients have been followed up during ten years, and they have shown radiological progression, and four are currently classified as PMF.

**Table 5:** Radiological classification and progression and their correspondent averaged Si and Al EDX detected percentages

Patients	CRX ILO classification*	Main HRCT findings*	CRX ILO classification^†^	Main HRCT findings^‡^	Si (%) ^ξ^	Al (%) ^ξ^	Progression rate per Year (PRY)
S1	SS 2/3 q/t	A,B,F	FMP A	A,B,E,D,F	0.07	0.0525	0.2727273
S2	PMF B	A,B,E	FMP C	A,B,E	0.595	0.165	0.0909091
S3	SS 2/2 r/q	A,B, D	FMP B	A,B,D,E	1.1325	0.3075	0.4545455
S4	SS 2/3 r/q	A,B,C	FMP C	A,B,D,E	1.1025	0.3075	0.4545455
S5	SS 3/2 r/q	A	SS 3/3 r/q	A,B	0.05	0.0875	0.0909091
S6	SS 1/2 q/q	A,B,C	SS 1/2 q/q	A,B	0.085	0.1725	0
S7	SS 2/2 q/t	A, B,	SS 2/2 q/t	A, B,	0.2775	0.0675	0

^*^At the time of lung biopsy. ^**†**^ Last follow-up visit. ^**‡**^Last HRCT. ^ξ^Averaged percentages of the EDX measurements at 4 regions (A to D) of each sample, obtained from the data in Table 4

*SS* Simple Silicosis, *PMF* Progressive Massive Fibrosis, *A* small nodular (round) opacities, *B* hilar and mediastinal lymphadenopathy, *C* ground-glass opacities, *D* Calcified lymphadenopathy, *E* Large opacities, *F* Interlobular septal thickening

Linear relationship between progression rate per year (PRY) and Si was statistically significant (r = 0.78, *p* = 0.03, IC 95% 0.07–0.97); therefore, the higher atomic content of Si in the nodule, the more probable progression of the illness. Linear relationship between PRY and Al was near significant (r = 0.71, *p* = 0.07, IC 95% -0.09–0.95).

In simple linear regression models, Si coefficient was statistically significant (*p* < 0.05). The coefficient estimate was 0.33, which means that an increase in one unit in the average atomic content of Si (%) increases the annualized progression rate by 0.33. However, it is not easy to discriminate the role of both in the progression of the disease because the correlation between Si and Al is very high (r = 0.93).

## Discussion

The Bay of Cadiz has suffered one of the largest known outbreaks of silicosis in ES workers. The first cases appeared in 2009, and although cases continue to appear today, the largest number of diagnoses occurred between 2010 and 2013 [11]. With this study, we aimed to elucidate if there may be factors other than silica, and inherent to the composition of countertops, that may be involved in the development of the disease.

The first objective of our study was to analyze the composition of the material with which our patients had been working in the years prior to diagnosis. As expected, the fundamental component found was silica, with percentages ranging from 87.2 to 99.5%, a result that is in agreement with the data presented by most authors [3, 12–14]. Among the SiO_2_ polymorphs found, two samples were formed almost entirely of cristobalite (M1 and M3), while quartz was the only component in two of the samples (M6 and M7); in other samples, similar percentages of both components were found.

Historically, cristobalite has been considered more harmful than quartz, but more recent studies have shown that the pathogenic effects are similar; consequently, the Occupational Safety and Health Administration (OSHA) has concluded that quartz, cristobalite and tridymite have similar toxic and carcinogenic potential [15].

In relation to the presence of noxious metallic elements in the samples, the complex and heterogeneous composition is noteworthy. Pavan and colleagues [3] detected, in addition to Si, up to 11 possible elements (Na, Mg, Al, S, K, Ca, Ti, Fe, Co, Cu, Zn); Al was present in different amounts in five of the samples. Di Benedetto and colleagues [12], in their samples, detected between 13 and 17 metals, in addition to Si, with a wide variability in the composition, with Ca and Na being the majority; others such as Mg, Al, K and Ti were frequently found in lower concentrations.

In this sense, samples M1 to M7 in our study showed to a greater or lesser extent the presence of 17 quantifiable metals. One of the elements systematically detected in all samples was tungsten. This has not been reported for any previous series, and we cannot rule out that the procedure used by us to obtain the dust samples could have led to W contamination (steel bits with tungsten carbide tips). The addition of elements contributing to ES dust has been verified by other authors who used similar tools in the processing of the material; the results can also differ based on dry or wet processing [12].

In our countertop samples, the metals found in the highest concentrations were Na, Ca, Al, Ti and Fe, accompanied by variable amounts of the other metals. Various types of metals have been implicated in the development of lung diseases of occupational origin. Chiba and colleagues described, on high resolution computerized tomography (HRCT), the presence of ground glass opacities, consolidations and centrilobular micronodules in a hard metal worker. Transbronchial biopsy exhibited chronic fibrotic and inflammatory changes, and the mineralogical analysis of lung tissue detected hard metals such as W, Ti and Fe, which were also detected in dust samples from the workplace [16]. Tomioka and colleagues described the existence of a micronodular pattern on HRCT and granulomatous lesions in the biopsy of a worker in an aluminum and battery processing factory [17]. In the analysis of the lung samples, the presence of silicon, iron, aluminum and titanium in the granulomas was confirmed. Aluminum, in particular, was distributed in a relatively high concentration in granulomatous lesions. Iijima and colleagues described ground glass opacities on HRCT, and on biopsy, bronchiocentric macrophage accumulation and peribronchiolar fibrosis were detected. The analysis found high amounts of aluminum and iron [18].

To our knowledge, the study presented herein is the first to analyze the distribution of Si and other metals in pulmonary nodules in ES silicosis. We obtained seven lung samples from the Anatomic Pathology files of two hospitals. In the analysis of our lung samples, we found that the pulmonary nodules have significant percentages of two fundamental exogenous elements, Si and Al, and that their atomic content was higher in the most central area of the nodule and decreased progressively in the periphery. This result highlights the significant accumulation of Al in nodules and is also a relatively abundant metallic element found in samples M1-M7 of countertops with which our patients worked prior to disease diagnosis. The higher concentration of Si and Al particles in the center of the nodules suggests that the cellular reactions begin at this point, precisely where the levels of silica and Al are higher.

The role of aluminum in silicosis has been historically controversial; in fact, from 1943 to 1979 thousands of miners were forced to inhale McIntyre Powder (a mixture of aluminum and aluminum hydroxide) [19] during years, for about 10 min before the underground work shift, with the aim to prevent the development of silicosis. As result, aluminum content in exposed miners’ lungs was three times higher than non-exposed [20]. The program ended in Canada in 1980 because it was concluded that this procedure granted no protective effect against silicosis [21].

Occupational exposure to Al has been associated with different histopathological patterns, including severe interstitial pulmonary fibrosis [22–24]. Assad and colleagues reported that aluminum inhalation can cause different phenotypes of interstitial lung disease, such as usual interstitial pneumonia or nodular pulmonary fibrosis [25]. It is possible that the differentiation of the interstitial lung disease phenotype in workers exposed to aluminum may depend on the type and intensity of exposure and the presence of concomitant exposures, such as silica, smoking and unknown host factors.

Abraham and Wiesenfeld described two cases of accelerated silicosis in sandblasting workers in which the mineralogical analysis of PMF lesions revealed not only silica but also aluminum silicate and other metals in significant amounts [26]. Halldin and colleagues studied the lung biopsy of a miner with category A PMF [27]. Of the 335 particles analyzed, 12.2% were silica, 82.4% were aluminum silicates, and 4% were titanium dioxide. The histopathological studies developed by Cohen and colleagues suggest that silica and silicates play an important role in the development of accelerated pneumoconiosis; however, in that study, no description was made of the metals that formed the silicates [28]. Jederlinic and colleagues described patients with pulmonary fibrosis, with more than 90% aluminum among the metals detected in the lung samples in workers exposed to alumina (Al_2_O_3_) [29], and Kraus and colleagues found ill-defined centrilobular nodular opacities detected by HRCT in 24.2% of aluminum powders workers [30]. Finally, Taiwo concluded that some evidence suggests that exposure to aluminum dusts and fumes may cause diffuse parenchymal changes, granulomas, pulmonary alveolar proteinosis and desquamative interstitial pneumonia[31]. One of our countertops samples (M2) contained 2.5% Aluminum (Al is contained in more complex albite compound), and Di Benedetto detected Al in all samples tested [12]. Aluminum, described as an elemental solid or as part of compounds or minerals, has been reported in other countertop samples [3, 13, 32].

Ophir and colleagues studied the composition of elements in the induced sputum of three workers exposed to processed ES and another three not exposed [33]. In both, different elements were found, but silicon, titanium, zinc and aluminum were only found in exposed patients. In this case, it was found that the Al concentration was similar to that of Si. The authors also studied samples of natural and artificial stone dust obtained from two industrial plants that worked exclusively on a single type of stone and found that the ES powder had 160 times more Si than that in powder obtained from a natural stone factory, and that the Al concentration was 22 times higher in ES powder than in natural stone. Studies on animals have shown that aluminum inhalation induces epithelial cell damage and an increase in collagen deposition and matrix metalloproteinases [34] and, on the other hand, it is known that aluminum salts, as aluminum hydroxide, increase the inflammatory response and induce the differentiation of monocytes into dendritic cells and macrophages. Aluminum particles are phagocyted by these cells (macrophages and dendritic cells), and they cause disruption of the phagolysosomes and release of active cathepsin B into the cytoplasm which may be a sufficient signal for NLPR3 (also named as NALP3) activation inflammasome, resulting in the release of active IL-1β and IL-18 [35, 36], and this is also a well-known pathogenic mechanism in silicosis [37, 38]. Furthermore, Al can induce DNA damage by oxidative stress or liberation of DNase [39]. Finally, Mandler and colleagues have demonstrated in mice that oropharyngeal aspiration of dust from a solid-surface composite composed of alumina trihydrate and acrylic polymer induced, in bronchoalveolar lavage fluid, a significant inflammatory response (lactate dehydrogenase activity, inflammatory cells, and pro-inflammatory cytokines), in some cases to a greater degree than the silica positive control. Histologically they found acute alveolitis at day 1 after exposure and alveolar particle deposition and granulomatous mass formation at day 14 [40].

The high proportion of Al in all our samples, in some cases with an Al/Si ratio higher than 1, raises the hypothesis that aluminum may play a role in ES silicosis, possibly enhancing the inflammatory response to silica or even causing an inflammatory reaction by itself.

Recently, Fireman and co-workers investigated silica and metals in lung tissues of patients that underwent lung transplantation due to ES silicosis and others due to Idiopathic Pulmonary Fibrosis. They found that only silica, titanium and aluminum were significantly higher in biopsies of ES silicosis patients [41]. Kα-X ray Ti peak is centered at 4.5 keV, within the range of our spectra (up to 5 keV). Therefore, if there is any Ti within the biopsies in our study, either it was a percentage below the detection threshold in the silicotic nodules, or the amount of Ti-containing particles was so low it passed undetected during the SEM study. A number of other exogenous elements, environmental and genetic factors different to Si and Al are of importance for the development of silicosis.

Although some researchers have studied the mineralogical and metallic composition of countertops, their organic components have only been partially studied [3]. This organic content can represent up to 14.3 wt.% of ES [32].These compounds are part of the resins used in manufacturing and reach the lungs as part of the dust particles inhaled during the working processes (cutting, drilling, polishing, etc.). Recently, Halls and colleagues have detected measurable concentrations of a wide range of VOCs emitted when cutting ES [42]. Although we do not know the pathogenic role of these compounds in ES workers, it should be taken into account that subchronic exposure to low-dose VOCs (formaldehyde, benzene, toluene, and xylene) produces bronchial and lung inflammation in animal models [43] and some low molecular weight PAHs, such as phenanthrene and fluorene, induce oxidative stress and inflammation in human lung epithelial cells and the combined action of both is more potent than individually [44].

On the other hand, styrene has been associated with bronchial asthma [45, 46] eosinophilic bronchitis [47], hypersensitivity pneumonitis and organizing pneumonia [48] and toluene affects respiratory system [49]. Toluene was detected in four of our countertop samples, while styrene was found in all of them. This could explain, then, that the spectrum of diseases related to manufacturing or manipulation of ES is broader than silicosis. Therefore, it would be necessary to take into account that work-related respiratory symptoms could be caused also by organic compounds and could explain the high rate of respiratory symptoms in ES workers without silicosis, which in some series affect up to one third of them [50].

The main limitation of our study is that the association found here does not, in itself, infer causality. Also, it can be argued that the number of biopsy samples is not high enough to support some conclusions, and so, the association founded between Al and Si may be a coincidence. Nevertheless, the fact that in all the studied samples Al and Si containing particles are found in significant amount merits further investigation. To the date, once the ES high power to produce silicosis is confirmed, the diagnosis, mostly, is not obtained through biopsy, but via exposure antecedents and chest radiography (CXR) or High-resolution CT (HRCT), thus, it has to be noted that obtaining lung samples for diagnosis is currently only justified in the cases of atypical presentations.

Until recently, exposure to ES was basically limited to its manufacture in large factories or to the completion and finishing of the product into bathroom and kitchen countertops by small companies. In such work environment, the continuous inhalation of this material has caused a multitude of cases worldwide, described by some as an epidemic [9], of which we are probably only seeing the tip of the iceberg [51]. This suspicion is reinforced by some studies; screening in ES workers in California has shown that 12% suffer from silicosis, diagnosed by chest radiology and ILO classification [52]. In addition, a more exhaustive evaluation in workers in Victoria (Australia) using chest radiology and HRCT showed that the percentage of patients with silicosis increased to 36% because HRCT detected cases with negative chest radiology [53]. If we consider that the number of exposed workers in the US is close to 100,000, we will have an idea of the magnitude of the problem. Therefore, it is necessary to find safer materials for workers, in addition to optimizing occupational prevention measures.

## Conclusions

In conclusion, some of the VOCs and PAHs that we have found in our samples of countertops has been described as causative of respiratory disease and lung inflammation. Within the silicotic nodules, the detected aluminum content can be considered high, and it is, in some cases, higher than the silicon one. Both exogenous elements decrease their content from the center to the frontier of the nodule. In any case, further studies are needed to clarify any specific role of the aluminum regarding the ES silicosis. Experimental studies with cell cultures and animal models with various types of ES with different compositions can help to more accurately define the potential aggressiveness of the different elements and organic compounds of ES.

## Materials and methods

The Spanish National Association of Silicosis-Affected and -Sick People (Asociación Nacional de Afectados y Enfermos de Silicosis—ANAES) was asked to send us samples of the countertops most frequently used by workers in our region in the years prior to the outbreak; we received seven representative samples of different models manufactured by two commercial companies.

The present study was performed on these seven solid samples (M1 to M7), and seven lung specimens of patients diagnosed previously with ES silicosis by lung biopsy (S1 to S7). Besides, one lung biopsy from an unexposed patient was used as a control sample.

### Preparation and analysis of the countertop samples:

**Fig. 4:**
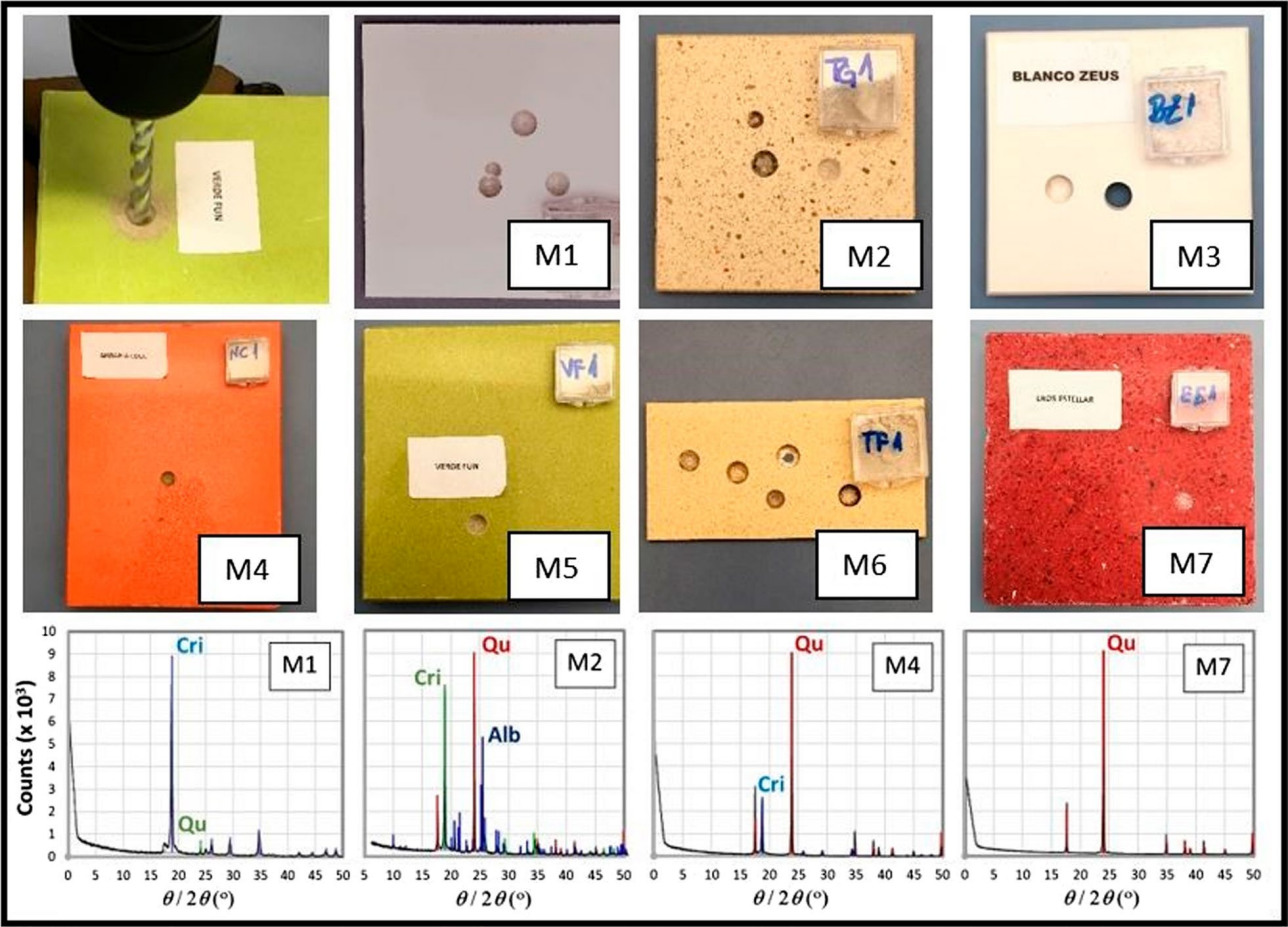
Images of the countertops analyzed, the drill bit used, and the powder extracted and 4 XRD diagrams for 4 of the samples. Among them, the major crystalline phases of SiO_2_, i.e., quartz (Qu) and cristobalite (Cri), were detected, as well as minerals containing Na (AlSi_3_O_8_), i.e., albite (Alb). In the sample M4, approximately 2% of crystals (other) comprised 0.7% SiO_2_ tridymite, 0.4% TiO_2_ rutile, 0.35% Pb_4_BiVO_8_, and 0.2% intermetallic Sn_3_Ti_5_

The mechanical disaggregation of each of the slabs (M1 to M7) was performed within an isolation hood in a laboratory area without environmental contaminants. The samples, in the form of fine powders, were generated from the friction of a 6-mm Wolfpack® professional widia drill bit using a low drilling speed until a sufficient amount of sample (2 cubic centimeters) was obtained. Figure 4 shows representative images of the countertop samples, the drill bit used and the powder extracted.

These powdery samples were processed using various procedures, which are described below, to obtain the complete composition and structure information of the crystalline and amorphous portions of each of the samples.

### Identification of crystalline phases and major compounds

Structural and compositional characterizations of each sample was performed through quantitative analysis by X-ray diffraction (XRD) using the q-XRD Rietveld Refinement method and peak ratio analysis (RIR: reference intensity ratio), allowing the determination of the crystalline and amorphous components in multiphase mixtures. The identification of the most frequent elements was performed by X-ray fluorescence (XRF) using qualitative and quantitative standardless methods. The studies were carried out with the equipment and technicians at the X-ray Division of the Central Services of Science and Technology (Servicios Centrales de Ciencia y Tecnología—SC-ICYT) of the University of Cadiz (UCA): Bruker D8Advance A25 Davinci, with a LINXEYE detector, for XRD and Bruker M4 Tornado for XRF.

### Analysis of metals

Seventeen metals were selected to be studied based on their harmfulness to humans or their possible presence in countertops. Detection was carried using the equipment and technicians of the Atomic Spectroscopy Division of SC-ICYT at UCA. Thermo Elemental—X7 Quadrupole inductively coupled plasma mass spectrometer (ICP-MS) was used for detection of As, Cd, Co, Cu, Ni, Pb, Zn, Ba, Mo, Sb, V, and Al, Fe, Ca, Na, Ti, W were detected using an Iris Intrepid Thermo Elemental inductively coupled plasma atomic emission spectrometer (ICP-AES). To prepare the samples, 100 mg of ES powder was digested with 5 mL of HF% (pa), 3 mL of 65% HNO and 1 mL of 30% H_2_O_2_ in an Ethos 1 microwave oven (Milestone, Italy) at 200 °C for 20 min to bring the sample to complete dissolution. The digestion products were filtered through a 0.45-μm nylon filter, and the samples were flushed with ultrapure water (resistivity less than 18.2 MΩ cm) to a final volume of 50 mL.

### Analysis of organic compounds

To each of the dust samples obtained from the countertops, a precise volume of specific solvents (carbon disulfide, acetonitrile, hexane and methanol, high-performance liquid chromatography grade) was added to a mass determined from the powder sample to extract and measure the organic portions of the polymeric binder of the countertops (EPA method 3570). The suspension was incubated, and the extraction of the different compounds considered a priori (target compounds) was achieved by ultrasonic bath. This suspension was filtered through a controlled porosity filter (0.2 μm) to remove solid particles. Then, the samples were processed for analysis by gas chromatography–mass spectrometry (GC–MS: Waters Synapt G2) coupled with a time-of-flight analyzer (high resolution) for the identification and quantification of volatile and semivolatile organic compounds (VOCs), polycyclic aromatic hydrocarbons (PAHs) and polychlorinated biphenyls (PCBs), aldehydes and ketones. For those compounds whose standards were available (PAHs, PCBs and some VOCs), identification and quantification were carried out with Masslynx software. The presence of characteristic masses (allowing an error of less than 5 ppm) and retention times were considered identification criteria to determine the concentrations in the different samples through corresponding calibration curves obtained for solvent-based standards [54]. The instrument provided linear responses in the range of concentrations up to 2000 ng/g (R2 higher than 0.985), for those samples which presented higher levels a dilution factor was applied to the sample extract. The limits of detection (LOD, defined as the lowest concentration providing a signal to noise ratio of 3) and quantification (LOQ, defined as the lowest concentration providing a signal to noise ratio of 10) were calculated (Table 6). The intra-day repeatability in the peak areas for a calibration standard injected two consecutive days was lower than 16%. For those compounds whose standard was not available (certain aldehydes, ketones, VOCs, etc.), the chromatographic peaks were compared with the mass spectra of the NIST (Standard Reference Database) library after analysis by GC–MS with electronic ionization (EI) and verification through the exact mass and elemental composition available in MassLynx software (unknown compounds or ‘nontarget’). The analysis was performed at the mass spectrometry division of SC-ICYT at UCA.

**Table 6:** Analytical parameters (limits of detection, limits of quantification and regression coefficient) for the proposed method

Compound	Type	R^2^	LOD (ng/g)	LOQ (ng/g)
Acenaphtene	PAH	0.993	0.97	3.22
Acenaphtylene	PAH	0.999	0.78	2.59
Anthracene	PAH	0.991	0.86	2.85
Benz(a)anthracene	PAH	0.985	0.48	1.62
Benzo(a)pyrene	PAH	0.985	1.93	6.44
Benzo(b + k)fluoranthene	PAH	0.987	1.39	4.63
Benzo(ghi)perylene	PAH	0.988	4.44	14.80
Chrysene	PAH	0.991	0.44	1.48
Dibenz(ah)anthracene	PAH	0.993	4.44	14.80
Fluoranthene	PAH	0.999	0.39	1.31
Fluorene	PAH	0.996	0.48	1.61
Indene(123-cd)pyrene	PAH	0.985	8.02	26.73
Naphthalene	PAH	0.996	2.48	8.25
Phenanthrene	PAH	0.997	0.91	3.04
Pyrene	PAH	0.998	0.37	1.22
PCB101	PCB	0.993	0.47	1.58
PCB138	PCB	0.989	0.98	3.28
PCB153	PCB	0.995	1.23	4.11
PCB180	PCB	0.990	1.65	5.51
PCB28	PCB	0.989	0.95	3.16
PCB52	PCB	0.990	0.36	1.20
1Hexanol 2 ethyl	COV	0.991	8.72	15.3
Styrene	COV	0.989	4.31	12.1
Toluene	COV	0.994	4.47	13.3
Ethylbenzene	COV	0.995	3.14	11.2
124 Trimethylbenzene	COV	0.995	2.72	9.25
1Methoxy 2 propanol	COV	0.987	4.65	15.68
n-butylacetate	COV	0.989	6.27	21.29
m-xylene	COV	0.985	3.32	10.35
p-xylene	COV	0.986	3.32	10.35

### Patients and biopsy samples

All patients had been diagnosed with ES silicosis previously at Puerta del Mar University Hospital and Puerto Real University Hospital (Cadiz, Spain) by lung biopsy. All the patients are currently followed up at our outpatient clinics. Electronic medical records, CXR, HRCT scans and histological samples were retrospectively reviewed. Chest radiographs were classified according to International Labour Office (ILO) criteria [55] by three trained readers.

### Preparation and study of histological samples

All histological samples were obtained from paraffin blocks from the Anatomic Pathology files of Puerta del Mar University Hospital and Puerto Real University Hospital (Cadiz, Spain). Samples taken by VATS were between 4.5 and 7 cm in length. Biopsies taken by BTB were between 1 and 2 mm in length. The number of biopsies by BTB ranged between one and four per patient. We studied all lung biopsies of our two hospitals diagnosed with silicosis that had worked with ES. Five-micron sections were cut from the paraffin blocks, and after staining the sections with hematoxylin-eosin, Periodic Acid-Schiff (PAS), and Ziehl-Neelsen, biopsies were studied by light microscopy.

Next, samples underwent a preparation process, following a standard protocol for biological samples [56], in order to be studied under an electron beam (which implies to be deposited in a vacuum chamber). First, a deparaffinization step was needed to remove the biologic material from the paraffin blocks (since otherwise SEM signals would come only from paraffin layers). Except for samples S6 and S7 (which size can be observed in Fig. 1), all biopsy samples presented lengths between 1 and 2 mm, and even lower depths. Therefore, from that point, a careful micromanipulation took place in order to avoid destroying or even bending or distorting the samples, since locating a microscopic area (nodule) in a millimetric-size sample using a SEM microscope might be highly time-consuming. Therefore, the micrographs obtained from light microscopy were utilized as road maps for locating the silicotic nodules in SEM microscopy, which imply avoid changing the shape of the sample from the way it was received in the paraffin block. Such ideas were taking into account throughout the following dehydration process (which removed the remaining paraffin in the samples using progressive concentrations of acetone) and during the final critical point process, which allow to refill the biological dehydrated tissue with CO_2_, so the SEM images correspond to a realistic image of this tissue before it was removed from the patient lung.

Once the samples were prepared, they were studied using a Zeiss-Auriga and a Nova-NanoSEM 450 (both with field effect guns as electron sources), working at 5 kV in order to obtain EDX signal while avoiding contamination deposition on the studied area. The first microscope is located at the Centro de Investigación, Tecnología e Innovación (CITIUS) facilities (Seville, Spain), while the second microscope is located at the Servicios Centrales de Investigación, Ciencia y Tecnología (SC-ICYT) facilities (Puerto Real, Spain).

## Statistical analysis

### Al and Si spatial concentrations

Differences in atomic content of Si and Al between regions at the center of the nodule (A), close to the center (B), periphery (C) and outside (D) the nodule were calculated with a Friedman test due to the common within-individual association and the lack of normality and homoscedasticity within each group. Shapiro–Wilk and Fligner-Killeen tests were used to contrast normality and homoscedasticity. Durbin-Conover post-hoc tests with Holm correction on p-values were carried out to contrast pairwise difference among nodule regions. Spatial relationship of the different regions A to D was not taken into account.

### Al and Si in disease progression

Statistical relationships between Al and Si atomic contents and the annual progression of silicosis were also studied.

As a mean to determine the relationships between Si and Al atomic contents and annualized illness progression, a linear assumption was made for ILO scale. This linearized scale considers every change in one ILO position as a one unit increase in severity. With this new variable, the progression rate per year (PRY) was defined as:

$$PRY=\frac{\Delta S}{t} (yea{r}^{-1})$$

where Δ*S* represents the number of positions in ILO scale that the patient has got worse over the follow-up time and *t* represents the number of years of follow-up.

Concentrations of Si and Al were calculated as averaged percentages of the EDX measurements at four regions (A to D) for each sample. Linear correlations between these two variables were estimated by Pearson correlation coefficient (r). Simple linear regressions were also performed with PRY as response variable and Al and Si concentrations as predictive variables. Normality of residuals was assessed using QQ-plots.

A value of *p* < 0.05 was established to determine statistical significance. All the analyses were performed using R software (R Core Team, 2021).

## Data Availability

All data and materials are included in the manuscript, tables and figures.

## Abbreviations

AS: Artificial stone
BSE: Backscattered electron
EDX: Energy-dispersive X-ray spectroscopy
ES: Engineered stone
GC-MS: Gas chromatography coupled to mass spectrometry
HRCT: High Resolution Computerized Tomography
ICP-AES: Inductively coupled plasma atomic emission spectrometer
ICP-MS: Inductively coupled plasma mass spectrometry
LOD: Limit of detection
LOQ: Limit of quantification
PCB: Polychlorinated biphenyls
PAH: Polycyclic aromatic hydrocarbons
PMF: Progressive massive fibrosis
ROS: Reactive oxygen species
SEM: Scanning electron microscopy
VOC: Volatile and semivolatile organic compounds
XRD: X-ray diffraction
XRF: X-ray fluorescence
